# Anticolon Cancer Effect of Korean Red Ginseng via Autophagy- and Apoptosis-Mediated Cell Death

**DOI:** 10.3390/nu14173558

**Published:** 2022-08-29

**Authors:** Kyoung Ah Kang, Cheng Wen Yao, Mei Jing Piao, Ao Xuan Zhen, Pincha Devage Sameera Madushan Fernando, Herath Mudiyanselage Udari Lakmini Herath, Seung Eun Song, Suk Ju Cho, Jin Won Hyun

**Affiliations:** 1 Department of Biochemistry, College of Medicine, Jeju National University, Jeju 63243, Republic of Korea; legna07@jejunu.ac.kr (K.A.K.); vane1989923@163.com (C.W.Y.); mjpiao@jejunu.ac.kr (M.J.P.); zhenaoxuan705@stu.jejunu.ac.kr (A.X.Z.); sameera@stu.jejunu.ac.kr (P.D.S.M.F.); lakmini@stu.jejunu.ac.kr (H.M.U.L.H.); 2 Jeju Research Center for Natural Medicine, Jeju National University, Jeju 63243, Republic of Korea; 3 Department of Anesthesiology, Jeju National University Hospital, College of Medicine, Jeju National University, Jeju 63241, Republic of Korea; dkrfnf@hanmail.net

**Keywords:** Korean red ginseng, colon cancer, autophagy, apoptosis, anticancer effect

## Abstract

Ginseng (*Panax ginseng* Meyer) has been used in East Asian traditional medicine for a long time. Korean red ginseng (KRG) is effective against several disorders, including cancer. The cytotoxic effects of KRG extract in terms of autophagy- and apoptosis-mediated cell death and its mechanisms were investigated using human colorectal cancer lines. KRG induced autophagy-mediated cell death with enhanced expression of Atg5, Beclin-1, and LC3, and formed characteristic vacuoles in HCT-116 and SNU-1033 cells. An autophagy inhibitor prevented cell death induced by KRG. KRG generated mitochondrial reactive oxygen species (ROS); antioxidant countered this effect and decreased autophagy. KRG caused apoptotic cell death by increasing apoptotic cells and sub-G_1_ cells, and by activating caspases. A caspase inhibitor suppressed cell death induced by KRG. KRG increased phospho-Bcl-2 expression, but decreased Bcl-2 expression. Moreover, interaction of Bcl-2 with Beclin-1 was attenuated by KRG. Ginsenoside Rg2 was the most effective ginsenoside responsible for KRG-induced autophagy- and apoptosis-mediated cell death. KRG induced autophagy- and apoptosis-mediated cell death via mitochondrial ROS generation, and thus its administration may inhibit colon carcinogenesis.

## 1. Introduction

Cancers are the leading cause of death globally and their treatment mainly involves surgery, chemotherapy, and radiotherapy [1]. Induction of apoptosis in cancer cells via anticancer drugs is thought to be effective for anticancer therapy [2]. However, the apoptosis pathway is abnormally regulated in human cancer cells, which may cause these cells to evade radiation and chemotherapy. Cancer cells escape death via resisting apoptosis, which upregulates antiapoptotic signals (e.g., Bcl-2, Akt, and Mcl-1) and downregulates proapoptotic signals (e.g., Bax, Bak, and Bad), thereby initiating and implicating faulty apoptosis [3,4]. The concurrent pharmacological strategy targeting autophagy and apoptosis signaling pathways might greatly improve the efficacy of anticancer therapy [5].

Autophagy is a phenomenon involving the degradation of cellular components and organelles in an autophagosome (double-membrane vesicle), which is transferred to and degraded in the lysosome [6]. Signaling pathways that are activated by chemotherapeutics and metabolic stress can stimulate autophagy [7]. After the induction of autophagy, cytoplasmic degradation of constituents in autolysosomes eventually leads to the formation of free nucleotides, amino acids, and fatty acids, which are recycled by the cell for nutrient reuse and energy regeneration. Autophagy can promote cell survival; however, it may also promote cell death [2,8], affording dual effects against cancer.

Cell death can occur through three distinct routes of cellular catabolism, namely apoptosis, autophagy, and necrosis. Among these three types of programmed cell death, autophagy-mediated cell death is morphologically defined as cell death without chromatin condensation but is accompanied by the formation of distinctive autophagosomes [9]. Although apoptosis and autophagy are considered as two different types of cell death, there is a link between them as they share common components that are regulated by common factors [2]. For instance, various molecular interactions between apoptosis and autophagy have been investigated in apoptosis-specific protein Beclin-1, the Bcl-2 protein family, Ca^2+^/calmodulin-dependent protein kinases, phosphatase and tensin homolog (PTEN), protein kinase B (Akt/PKB), and mammalian target of rapamycin (mTOR) [5,10]. Therefore, in particular situations, many signaling pathways regulate autophagy, which can also induce apoptosis to cause cell death.

Reactive oxygen species (ROS) have been involved in the signal pathways of cellular apoptosis and autophagy [11]. It has been reported that high levels of glycation end products increase cellular apoptosis and autophagy via the ROS-mediated extracellular signal-regulated protein kinases (ERK) signaling pathway [12]. Several anticancer drugs eventually trigger autophagy-mediated cell death via various molecules and signaling pathways [5]. For instance, tanshinone IIA exerts its autophagy-inducing effect against oral squamous cell carcinoma by inducing the Beclin-1/Atg7/Atg12-Atg5 pathway and suppressing the PI3K/Akt/mTOR survival pathway [13]. A dietary phenolic compound, fisetin, increases autophagy- and apoptosis-mediated cell death via activation of ERK1/2 and attenuation of Akt/NF-κB/mTOR signaling pathways [14].

Ginseng (*Panax ginseng* Meyer) has been utilized in East Asian traditional medicine for a long time. Red ginseng, which is tinted reddish-brown, is prepared by steaming and then drying [15]. Korean red ginseng (KRG) is effective against several disorders, including cancer [15]. KRG extract has been shown in in vivo and in vitro studies to exert anticancer effects in different types of cancer. Oral administration of KRG extract significantly decreases the incidence of lung adenoma and liver cancer [16]. It prevents gastric cancer caused by *Helicobacter pylori* infection by inhibiting H_2_S-induced angiogenesis [17]. It was also shown to inhibit carcinogen-induced mouse skin papilloma and decrease the number of tumors in each mouse [18]. In addition, in colon cancer, KRG extract inhibits hypoxia-induced epithelial-to-mesenchymal transition and invasion via the NF-κB and ERK1/2 pathways [19]. However, the effects and mechanisms of action of KRG extract on human colon cancer are not known in detail. In this study, mechanisms of KRG extract-induced cell death were investigated in terms of autophagy- and apoptosis-mediated cell death in human HCT-116 and SNU-1033 colon cancer cells.

## 2. Materials and Methods

### 2.1. Reagents

Ginsenosides (Rb1, Rc, Rg2, and Rg3), 3-(4,5-dimethylthiazol-2-yl)-2,5-diphenylte-trazolium bromide (MTT; cat. M5655), z-VAD-FMK (cat. V116), bafilomycin A1 (cat. B1793), *N*-acetyl-l-cysteine (NAC; cat. A9165), and propidium iodide (PI; cat. P4170) were purchased from Sigma-Aldrich (St. Louis, MO, USA). Primary antibodies against Atg5 (cat. sc-515347), Bcl-2 (cat. sc-7382), caspase-9 (cat. sc-133109), caspase-3 (cat. sc-7148), poly (ADP-ribose) polymerase 1 (PARP1; cat. sc-8007), and actin (cat. sc-376421) were purchased from Santa Cruz Biotechnology (Santa Cruz, CA, USA). The LC3 (cat. 2775), Beclin-1 (cat. 3738), and phospho-Bcl-2 (cat. 2827) antibodies were purchased from Cell Signaling Technology (Beverly, MA, USA). Dihydrorhodamine (DHR) 123 (cat. D23806) and acridine orange (cat. A3568) were obtained from Molecular Probes (Eugene, OR, USA) and Invitrogen (Carlsbad, CA, USA), respectively.

### 2.2. KRG Preparation

KRG extract from the roots of 6-year-old *P. ginseng* was prepared by the Korea Ginseng Corporation (Seoul, Korea) as described previously [20]. Briefly, P. ginseng roots were steamed at 90–100 °C for 3 h, and then dried at 50–80 °C. Next, the KRG extract was isolated by circulating water at 85–90 °C three times for 8 h. The ginsenosides in the KRG extract were compared with ginsenoside standards using ultra-performance liquid chromatography. The KRG extract contained Rb1 (4.62 mg/g), Rb2 (1.83 mg/g), Rc (2.41 mg/g), Rd (0.89 mg/g), Re (0.93 mg/g), Rf (1.21 mg/g), Rg1 (0.71 mg/g), Rg2 (3.21 mg/g), and Rg3 (3.05 mg/g) as the major ginsenosides. The KRG extract powder was dissolved in dimethyl sulfoxide (DMSO; stock solution 100 mg/mL).

### 2.3. Cell Culture

The human colon cancer cell lines HCT-116, SNU-1033, Caco-2, and HT-29 were purchased from the Korean Cell Line Bank (Seoul, Korea), and normal human colon FHC cells were obtained from the American Type Culture Collection (Rockville, MD, USA). HCT-116, SNU-1033, and HT-29 cells were maintained in RPMI 1640 medium (Gibco, Life Technologies, Grand Island, Carlsbad, CA, USA) supplemented with 10% fetal bovine serum (FBS) (Gibco), and Caco-2 cells were cultured in minimum essential medium (Gibco) supplemented with 10% FBS. FHC cells were cultured in a 1:1 mixture of Ham’s F12 and Dulbecco’s modified Eagle’s medium (Gibco) containing 10% FBS, HEPES (25 mM; Gibco), cholera toxin (10 ng/mL; Sigma-Aldrich), insulin (5 μg/mL; Sigma-Aldrich), transferrin (5 μg/mL; Sigma-Aldrich), and hydrocortisone (100 ng/mL; Sigma-Aldrich).

### 2.4. Cell Viability

Cytotoxicity of KRG or ginsenosides (Rb1 Rc, Rg2, and Rg3) against various colon cancer cells and normal colon cells was evaluated using the MTT assay, as previously described [21]. Cells were seeded (1.5 × 10^5^ cells/well) in 96-well plates and incubated with the KRG extract or ginsenosides for 48 h at various concentrations. For inhibitor or NAC treatment, cells were seeded (1.5 × 10^5^ cells/well) and after pre-treatment with the inhibitors (z-VAD-FMK; 5 µM or bafilomycin A1; 1 µM) or NAC (1 mM) for 1 h, they were treated with KRG for 48 h. After incubation with MTT solution for 4 h, the formazan crystals formed were dissolved in DMSO. The absorbance of the formazan product was read via a scanning multi-well spectrophotometer at 540 nm [21].

### 2.5. Acridine Orange Staining

For detection of acidic intracellular compartments, acridine orange staining was performed. Acridine orange in acidic vesicles (autolysosomes) emits bright red fluorescence; it is protonated and forms aggregates, whereas acridine orange in the nucleus emits bright green fluorescence [22,23]. For confocal image analysis, colon cancer cells were seeded (0.8 × 10^5^ cells/mL) in chamber slides and incubated at 37 °C for 16 h. The cells were treated with KRG (at IC_50_ value for each cell line) for 48 h. The cells were stained with 10 μg/mL acridine orange at 37 °C for 30 min and observed under a laser scanning confocal microscope (Carl Zeiss, Jena, Germany). For FACS analysis, colon cancer cells were seeded (0.8 × 10^5^ cells/mL) in 6-well plates and incubated at 37 °C for 16 h. The cells were treated with KRG (at IC_50_ value for each cell line) for 48 h. Thereafter, the cells were stained with 10 μg/mL acridine orange and red fluorescence was detected using a FACSCalibur™ flow cytometer (Becton Dickinson, Mountain View, CA, USA), separately. Autophagic lysosomes fluoresced orange or red within cytoplasmic vesicles, whereas nuclei fluoresced green.

### 2.6. LC3 Transfection and Measurement of LC3-Positive Puncta

LC3 is recruited to autophagosomes, forming punctate structures [24]. The cells were transfected with a green fluorescent protein (GFP)-tagged LC3-expressing plasmid (CBA401; Cell Biolabs, San Diego, CA, USA) using Lipofectamine™ 2000 reagent (Invitrogen) according to the manufacturer’s instructions. The suitable cell confluency in a 60 mm culture dish was 80–90% at the time of transfection. GFP-LC3-expressing plasmid (5 μg/mL) was used for transfection of cells in a culture dish. Lipofectamine™ 2000 reagent and plasmid were freshly diluted to equal volumes with Opti-MEM^®^ (Gibco 31985070; Invitrogen) medium prior to use. The plasmid/Lipofectamine mixtures were incubated for 15 min at room temperature; the growth medium was replaced by Opti-MEM^®^ medium and then the mixtures (1000 μL for 60 mm culture dish) were added. After incubation at 37 °C for 4 h, the growth medium was changed, and the cells were incubated with the KRG extract for 48 h. The fluorescence of GFP-LC3 was then detected using a laser scanning confocal microscope (Carl Zeiss).

### 2.7. Western Blot Analysis

The expression levels of proteins associated with cell apoptosis and autophagy were detected using western blot analysis. Cells were seeded (1.5 × 10^5^ cells/well) in 100 mm culture dish and incubated with the KRG extract for 48 h. Total protein was extracted from the cells using the RIPA lysis buffer (Thermo Fisher, Life Technologies, Grand Island, Carlsbad, CA, USA), containing protease inhibitors (Thermo Fisher), according to the manufacturer’s instructions. Extracted proteins were subjected to SDS-PAGE, and the separated proteins were then transferred from the gel onto nitrocellulose membranes. After blocking with 10% BSA for 1 h, the membranes were incubated with primary antibodies against Atg5, Beclin-1, phospho-Bcl-2, Bcl-2, caspase-9, caspase-3, PARP1, LC3, and actin, and then horseradish peroxidase-conjugated immunoglobulin G secondary antibodies (Pierce, Rockford, IL, USA). Incubation with primary and secondary antibodies was carried out for 2 h at room temperature. Protein bands were detected using the Amersham ECL western blotting Detection Reagent (GE Healthcare Life Sciences, Buckinghamshire, UK).

### 2.8. Detection of Sub-G_1_ Hypodiploid Cells

To determine the apoptotic effect of KRG, flow cytometry was performed using propidium iodide (PI) staining of cells. The cells were seeded (0.8 × 10^5^ cells/mL) in 6-well plates and incubated at 37 °C for 16 h. The cells were treated with KRG (at IC_50_ value for each cell line) for 48 h. These cells were fixed with 70% ethanol for 30 min at 4 °C and treated with PI and RNase A for 30 min at 37 °C. The PI-stained cells were evaluated with a FACSCalibur™ flow cytometer (Becton Dickinson) and the sub-G_1_ hypodiploid cells were analyzed via histogram data assessed using the CellQuest™ and ModFit software.

### 2.9. Mitochondrial ROS Measurement

To determine the ROS levels in mitochondria, the cells were seeded (0.8 × 10^5^ cells/mL) in 6-well plates and after treatment with NAC (1 mM) for 1 h, they were treated with KRG for 48 h. After the cells were reacted with 20 μM DHR 123 for 30 min at 37 °C, images of the stained cells were observed using a laser scanning confocal microscope (Carl Zeiss). The DHR 123-stained cells were also analyzed using a flow cytometer and a fluorospectrometer.

### 2.10. Transfection with Short-Interfering RNA (siRNA)

To determine the role of Beclin-1 in KRG-treated colon cancer cells, we performed transient knockdown of Beclin-1 using siRNA transfection for various studies. The transfection was performed in 1000 μL of Opti-MEM^®^ medium with 2 μL of Lipofectamine^®^ RNAiMAX (Thermo Fisher 13778100, Waltham, MA, USA), and 10–50 nM siRNA per 60 mm culture dish. After 30 min, the mixture was added to the cells cultured at 37 °C in a CO_2_ incubator for 24 h. The siRNAs used for transfection were mismatched siRNA control (Control siRNA-A; sc-37007, sequence not disclosed by the manufacturer; Santa Cruz Biotechnology) and siRNA against Beclin-1 (siBeclin-1; Bioneer Inc., Daejeon, Korea; sense, 5′-ACUUUGCUGUAACCCUGUA (dTdT)-3′; antisense, 5′-UACAGGGUUACA GCAAAGU (dTdT)-3′).

### 2.11. Hoechst 33342 Staining

To measure the formation of apoptotic bodies, the cells were seeded (0.8 × 10^5^ cells/mL) in 6-well plates and incubated at 37 °C for 16 h. The cells were then treated with KRG or ginsenosides for 48 h. The treated cells were stained with Hoechst 33342 dye (Sigma-Aldrich) for 10 min at 37 °C, and images were captured using a fluorescence microscope equipped with a CoolSNAP-Pro color digital camera (Media Cybernetics, Rockville, MD, USA). Apoptotic cells were regarded as cells with Hoechst 33342-stained bright and fragmented nuclei. The numbers of cells in both viable and apoptotic single cells were measured. The index of apoptotic cells was determined as the apoptotic single cells/total cells (viable and apoptotic single cells).

### 2.12. Immunoprecipitation

Immunoprecipitation (IP) was performed to detect the interaction of Bcl-2 with Beclin-1. Cell lysates were collected and immunoprecipitated using an anti-Beclin-1 antibody in an immunoprecipitation buffer, and subsequently incubated with an agarose-conjugated secondary antibody. Western blotting was performed using anti-Bcl-2 to detect the association of Beclin-1 and Bcl-2 [25].

### 2.13. Statistical Analysis

Results are shown as means ± standard errors of the mean (SEM). The Sigma Stat software v12 (SPSS, Chicago, IL, USA) was used for statistical analyses. The data were examined using a one-way analysis of variance (ANOVA) followed by Tukey’s post-hoc test. A value of *p* < 0.05 was considered statistically significant.

## 3. Results

### 3.1. Selective Induction of Cell Death by KRG Extract in Colon Cancer Cells

**Figure 1:**
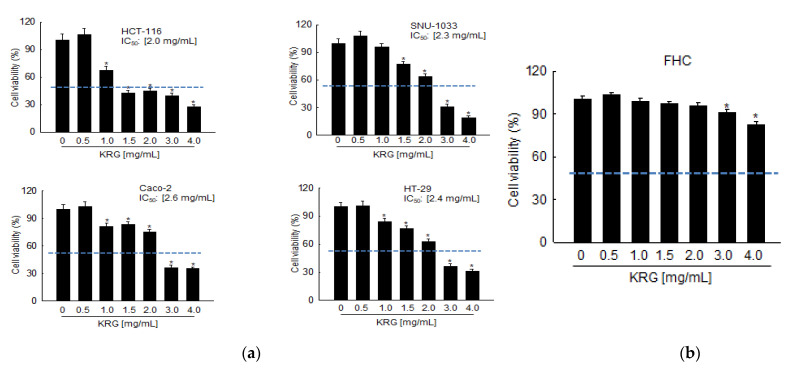
KRG extract induces cell death in colon cancer cells. Cell viability following KRG extract treatment for 48 h was assessed using the MTT assay (**a**) in HCT-116, SNU-1033, Caco-2, HT-29, and (**b**) FHC cells. The IC_50_ value of each cancer cell line is presented. The blue dot line indicates 50% of cell viability. * *p* < 0.05 vs. untreated control.

Cell viability was measured after incubating various colon cancer cells for 48 h with various concentrations of the KRG extract. As shown in Figure 1a, the KRG extract suppressed cell viability in a concentration-dependent manner, with 50% growth inhibition (IC_50_) observed at 2.0 mg/mL for HCT-116, at 2.3 mg/mL for SNU-1033, at 2.6 mg/mL for Caco-2, and at 2.4 mg/mL for HT-29 cells. When the IC_50_ values for these four cell lines were compared, the KRG extract was found to be more effective against HCT-116 and SNU-1033 cells; therefore, all subsequent experiments were conducted using HCT-116 and SNU-1033 cells. In contrast to these colon cancer cells, the KRG extract at 2.0 mg/mL did not show cytotoxic effects against FHC cells, which are normal non-transformed colon cells (Figure 1b), signifying that the KRG extract was selectively cytotoxic toward cancer cells.

### 3.2. Autophagy-Mediated Cell Death Induced by KRG Extract

**Figure 2:**
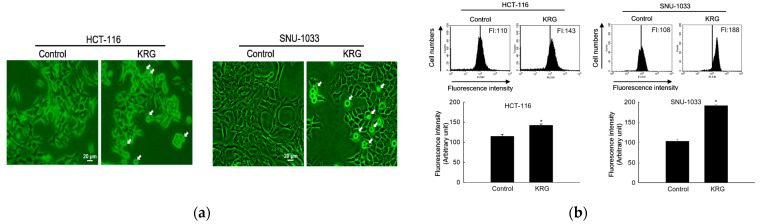

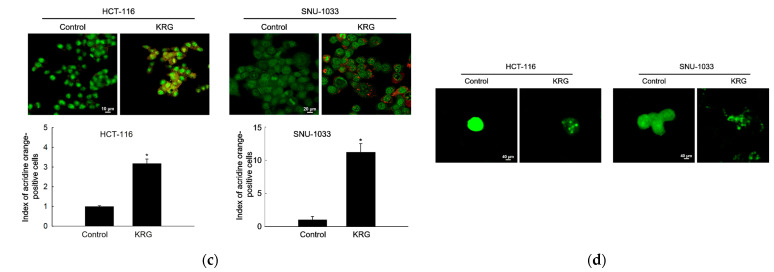

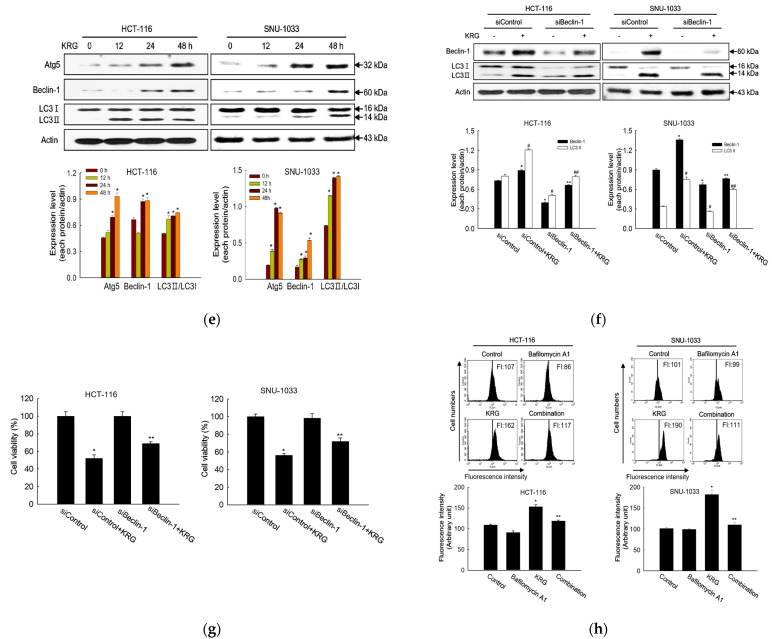

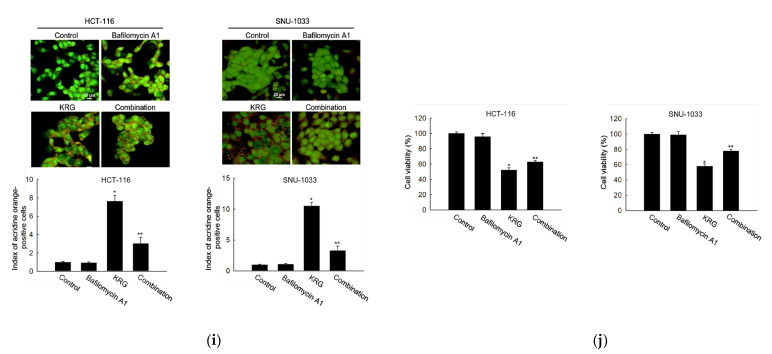
KRG extract induces autophagy-mediated cell death. (**a**) Light microscopy images of cells after 48 h of treatment with the KRG extract (2.0 mg/mL in HCT-116; 2.3 mg/mL in SNU-1033). The vacuolated cells are indicated by arrows. Scale bar, 20 μm. (**b**) Flow cytometry analysis and (**c**) fluorescence microscopy analysis after staining of cells with acridine orange. * *p* < 0.05 vs. untreated control. (**d**) Fluorescence microscopy analysis of GFP-LC3-transfected cells. Scale bar, 40 μm. (**e**) Levels of Atg5, Beclin-1, LC3-I, and LC3-II in the KRG extract-treated cells were assessed using Western blot analysis. * *p* < 0.05 vs. 0 h group. (**f**) After transfection of cells with siRNA against Beclin-1, the expression levels of beclin-1, LC3-I, and LC3-II were determined using western blot analysis. For Beclin-1 expression, * *p* < 0.05 vs. untreated siControl; ** *p* < 0.05 vs. the KRG extract-treated siControl cells. For LC3-II expression, ^#^ *p* < 0.05 vs. untreated siControl; ^##^ *p* < 0.05 vs. the KRG extract-treated siControl cells. (**g**) Cell viability was measured using the MTT assay. * *p* < 0.05 vs. untreated siControl; ** *p* < 0.05 vs. the KRG extract-treated siControl cells. Cells were incubated with the KRG extract (2.0 mg/mL in HCT-116; 2.3 mg/mL in SNU-1033) without or with bafilomycin A1. Autophagy was detected using (**h**) flow cytometry and (**i**) fluorescence microscopy after staining the cells with acridine orange. Scale bar, 10 μm (HCT-116) and 20 μm (SNU-1033). * *p* < 0.05 vs. untreated control; ** *p* < 0.05 vs. the KRG extract-treated cells. (**j**) Cell viability was evaluated using the MTT assay. * *p* < 0.05 vs. untreated control; ** *p* < 0.05 vs. the KRG extract-treated cells.

Microscopic observation showed the formation of vacuolated cells, indicating autophagy in HCT-116 and SNU-1033 cells after treatment with 2.0 and 2.3 mg/mL KRG extract for 48 h (Figure 2a). Therefore, we performed flow cytometry analysis using fluorescent acridine orange dye, a lysosomal marker that can stain acidic organelles, including lysosomes and vacuoles. As shown in Figure 2b, acridine orange fluorescence intensity was higher in the KRG extract-treated cells than in control cells. This result was further confirmed by confocal microscopy (Figure 2c). Autophagy is identified by the formation of autophagosomes, which are marked by the presence of membrane-bound LC3-phospholipid conjugates. GFP-LC3-transfected cells showed that the KRG extract treatment increased the abundance of punctate GFP-LC3 structures in the cells (Figure 2d). The KRG extract also increased the levels of LC3-II (the 14 kDa, processed form of LC3) with Atg5 and Beclin-1 expression in a time-dependent manner (Figure 2e). Furthermore, siBeclin-1 effectively attenuated the KRG extract-induced production of LC3-I and its subsequent conversion to LC3-II (Figure 2f), as well as cell death induced by the KRG extract (Figure 2g). These data indicate that the KRG extract significantly induced autophagy via Atg proteins. In addition, bafilomycin A1, an inhibitor of the late phase of autophagy, attenuated the accumulation of the KRG extract-induced acridine orange-positive vacuoles (Figure 2h,i) and cell death (Figure 2j).

### 3.3. Apoptosis-Mediated Cell Death by KRG Extract

**Figure 3:**
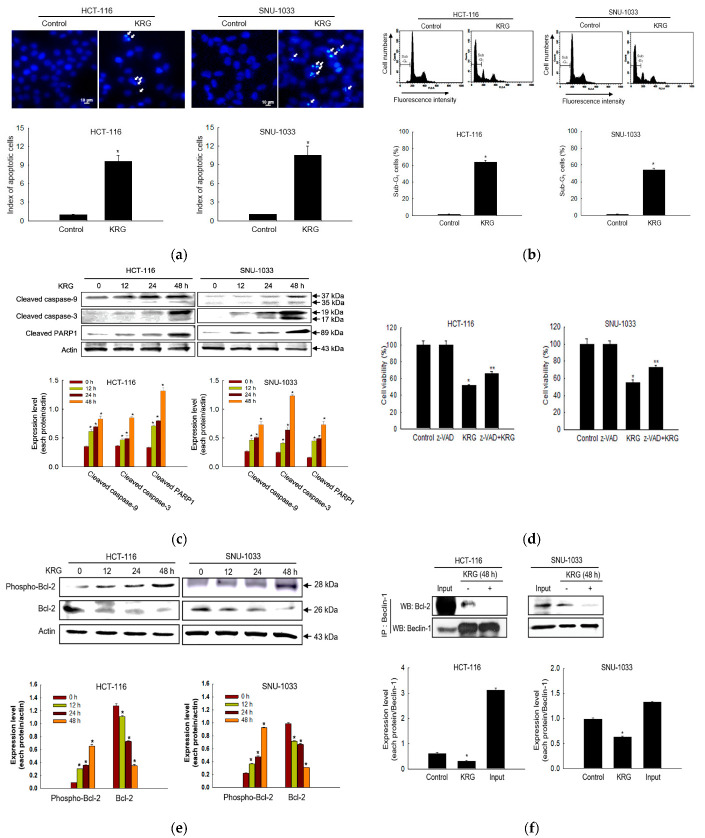
KRG extract induces apoptosis. The KRG extract-treated cells were incubated for 48 h or for the indicated durations. (**a**) The formation of apoptotic cells was assessed and quantified after staining of the cells with Hoechst 33342. Apoptotic cells are indicated by arrows. Scale bar, 10 μm. * *p* < 0.05 vs. untreated control. (**b**) Apoptotic sub-G_1_ DNA content was detected and quantified using flow cytometry after PI staining. * *p* < 0.05 vs. untreated control. (**c**) Active (cleaved) caspase-9 (35 and 37 kDa), caspase-3 (17 and 19 kDa), and PARP1 (89 kDa) levels were assessed using western blot analysis. * *p* < 0.05 vs. 0 h group. (**d**) Cells were incubated with the KRG extract (2.0 mg/mL in HCT-116; 2.3 mg/mL in SNU-1033) in the absence or presence of z-VAD-FMK (5 μM) for 48 h, and cell viability was assessed using the MTT assay. * *p* < 0.05 vs. control; ** *p* < 0.05 vs. the KRG extract-treated cells. (**e**) Bcl-2 and phospho-Bcl-2 levels were assessed using western blot analysis. * *p* < 0.05 vs. 0 h group. (**f**) Cell lysates were subjected to immunoprecipitation with an antibody against Beclin-1, and western blot analysis was performed with an antibody against Bcl-2. Input is the whole cell lysate and the untreated group is the immunoprecipitate of the cell lysate with Beclin-1 antibody. * *p* < 0.05 vs. untreated control.

The KRG extract induced apoptosis, as evidenced by the formation of apoptotic cells (Figure 3a), an increase in the number of sub-G_1_-phase cells (Figure 3b), and an increase in the levels of cleaved caspase-9, caspase-3, and PARP1 (Figure 3c) in colon cancer cells. In addition, the KRG extract-mediated apoptotic cell death was partially prevented by z-VAD-FMK, a pan-caspase inhibitor (Figure 3d). The KRG extract increased the phospho-Bcl-2 in a time-dependent manner with a concomitant decrease in the Bcl-2 level (Figure 3e). The interaction of Beclin-1 and Bcl-2 was decreased in the KRG extract-treated cells (Figure 3f).

### 3.4. Autophagy-Mediated Cell Death by KRG Extract via ROS Production

**Figure 4:**
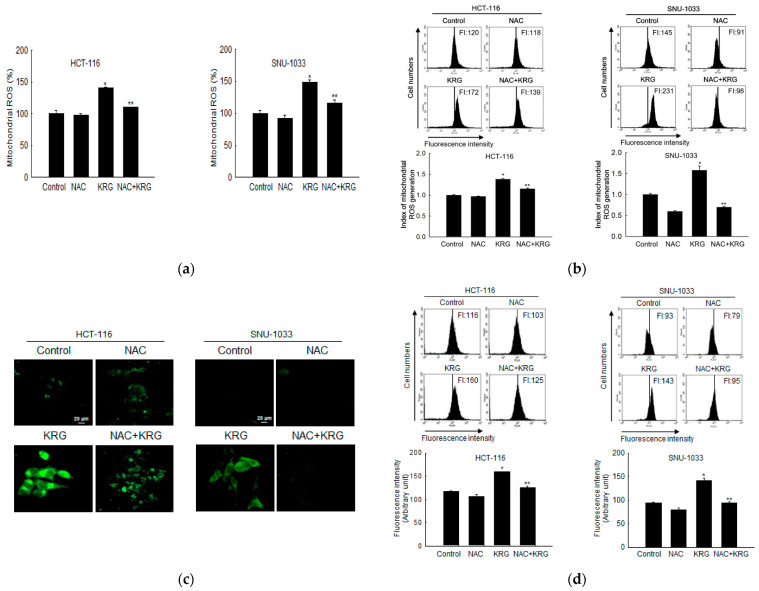

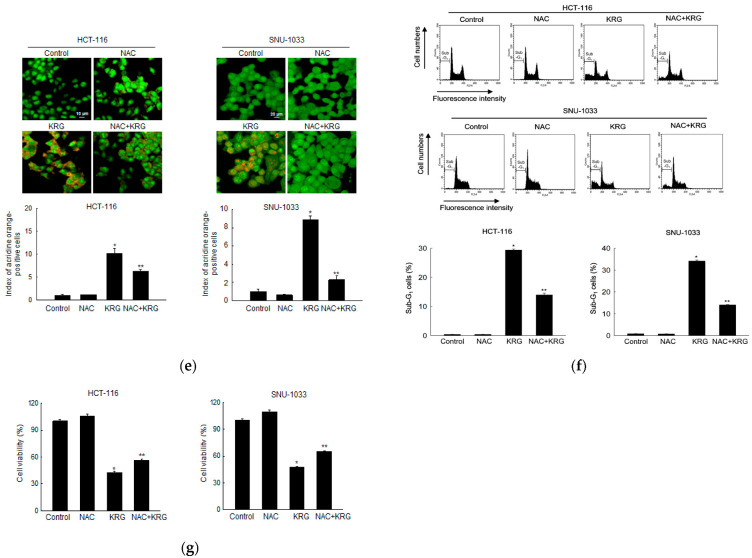
KRG extract induces autophagy-mediated cell death via generation of mitochondrial ROS. Cells were incubated with the KRG extract (2.0 mg/mL in HCT-116; 2.3 mg/mL in SNU-1033) in the presence or absence of NAC (1 mM) for 48 h. Mitochondrial ROS levels were assessed using (**a**) spectrofluorometry, (**b**) flow cytometry, and (**c**) fluorescence microscopy after DHR 123 staining. Scale bar, 20 μm. * *p* < 0.05 vs. untreated control; ** *p* < 0.05 vs. the KRG extract-treated cells. The KRG extract-induced autophagy was assessed using (**d**) flow cytometry and (**e**) fluorescence microscopy after acridine orange staining. Scale bar, 10 μm (HCT-116) and 20 μm (SNU-1033). * *p* < 0.05 vs. untreated control; ** *p* < 0.05 vs. the KRG extract-treated cells. (**f**) Apoptotic sub-G_1_ DNA content was detected and quantified using flow cytometry after PI staining. * *p* < 0.05 vs. untreated control; ** *p* < 0.05 vs. the KRG extract-treated cells. (**g**) Cells were incubated with the KRG extract (2.0 mg/mL in HCT-116; 2.3 mg/mL in SNU-1033) in the presence or absence of NAC (1 mM) for 48 h, and cell viability was assessed using the MTT assay. * *p* < 0.05 vs. control; ** *p* < 0.05 vs. the KRG extract-treated cells.

To evaluate whether autophagy-mediated cell death can occur through mitochondrial ROS, the generation of ROS was assessed using DHR 123 staining in the KRG extract-treated cells, followed by spectrofluorometry, flow cytometry, and confocal microscopy. The KRG extract induced the generation of mitochondrial ROS in colon cancer cells (Figure 4a,b). However, the enhanced mitochondrial ROS level was markedly attenuated by 1 mM NAC, an antioxidant. This result was also confirmed using confocal microscopy (Figure 4c). NAC attenuated the KRG extract-induced autophagy, as evidenced by the reduced number of acridine orange-stained cells (Figure 4d,e). In addition, NAC attenuated the KRG extract-induced increase in the number of sub-G_1_-phase cells (Figure 4f) and cell viability (Figure 4g). These results suggest that ROS generation induced by treatment with the KRG extract was involved in autophagy and cell death induction.

### 3.5. Ginsenoside Rg2 of KRG Is the Most Effective Component Responsible for KRG-Induced Autophagy-Mediated Cell Death or Apoptosis of Colon Cancer Cells

**Figure 5:**
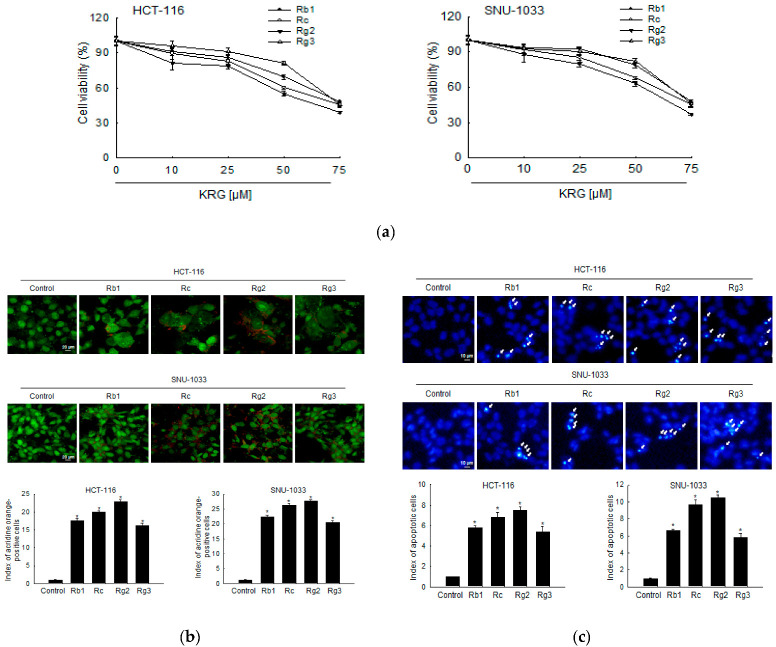
Ginsenoside Rg2 of KRG is the most effective component responsible for KRG-induced autophagy- and apoptosis-mediated cell death of colon cancer cells. (**a**) HCT-116 and SNU-1033 cells were treated with Rb1, Rc, Rg2, and Rg3 for 48 h, and an MTT assay was assessed. HCT-116 and SNU-1033 cells were treated at 50 μM of Rb1, Rc, Rg2, and Rg3 for 48 h. (**b**) Autophagy was analyzed using fluorescence microscopy after staining with acridine orange. Scale bar, 20 μm. * *p* < 0.05 vs. untreated control. (**c**) The formation of apoptotic cells was assessed and quantified after staining of the cells with Hoechst 33342. Apoptotic cells are indicated by arrows. Scale bar, 10 μm. * *p* < 0.05 vs. untreated control.

We evaluated ginsenosides Rb1, Rc, Rg2, and Rg3, which are the top four most abundant ginsenosides in the KRG extract, for their effects on autophagy-mediated cell death or apoptosis of colon cancer cells. As shown in Figure 5a, ginsenosides suppressed the proliferation of HCT-116 and SNU-1033 cells in a concentration-dependent manner, and Rg2 was found to be the most effective in both the cell lines. The fluorescence intensity of acridine orange in ginsenoside Rg2-treated cells was higher than that in other ginsenoside-treated cells (Figure 5b). In addition, ginsenoside Rg2 was the most effective inducer of apoptosis among the ginsenosides, as indicated by the formation of apoptotic bodies (Figure 5c).

## 4. Discussion

Ginseng is a valuable agricultural commodity grown for use in many traditional medicinal therapies. KRG is a type of ginseng preparation that is produced by steaming and drying fresh ginseng [26]. Ginseng saponin consists of triterpenoid glycosides of dammarane containing glucose, arabinose, xylose, and rhamnose [27]. Ginsenosides, the important bioactive components in ginseng, possess a common triterpene backbone and exhibit structural diversity. To date, about 200 ginsenosides have been isolated from various strains of ginseng. These include 20(S)-ginsenoside-Rg3, ginsenoside-Rh2, Rs1, Rs2, Rs3, Rs4, and Rg5; notoginsenoside-R4 in the protopanaxadiol group; and 20(R)-ginsenoside-Rh1, ginsenoside-Rh4 and F4 in the protopanaxatriol group [27]. Moreover, some ginsenosides, such as Rg2, Rg3, and Rh1, exist in different stereoisomeric forms, 20-(S) and 20-(R), depending on the position of the hydroxyl group in C-20. Other ginsenosides, including Rb2, Rb3, Rc, aglycone, Rg1, and F11, contain different saccharide substituents [28]. Ginsenosides have various pharmaceutical activities [29]. For example, CK, Rd, Rg2, Rg3, and Rg5 ginsenosides exhibit their anticancer effects by inhibiting differentiation, proliferation, and metastasis and induce autophagy, apoptosis, and cell cycle arrest by altering pathways related to carcinogenesis in breast, brain, lung, liver, and gastric cancer cell lines [29].

Ginsenoside Rg2 is the major component of KRG. The pharmacological properties of Rg2 have been demonstrated in many in vitro and in vivo studies. Rg2 has a role in inhibiting hepatic glucose production in HepG2 cells; this is achieved by the activation of the AMP-activated protein kinase pathway [30]. Rg2 has also been found to have a neuroprotective effect on glutamate-induced neurotoxicity, a result of mechanisms related to antiapoptosis and antioxidation [31]. Moreover, it has been demonstrated that Rg2 mediates anticancer effects by activating cell cycle arrest and signaling pathways related to mitochondrial damage-induced ROS production and apoptosis [32].

We showed, using our system, that the KRG extract exerts cytotoxicity in human colon cancer cells by triggering both autophagy- and apoptosis-mediated cell death pathways via ROS production. Although autophagy has a dual effect in facilitating cell survival and cell death, it was confirmed that autophagy induced by the KRG extract triggered cell death in HCT-116 and SNU-1033 cells, rather than survival, as observed in our cell viability assays using siBeclin-1-transfected cells and bafilomycin A1, a specific autophagy inhibitor. Despite the distinct characteristics of autophagy- and apoptosis-mediated cell death, many connections between them have been identified. Molecular mediators involved in apoptosis simultaneously trigger autophagy-mediated cell death. For instance, p53, which is a famous transcription factor that regulates apoptosis and cell cycle progression by promoting cyclin-dependent kinase inhibitors (e.g., p16 and p21) and active caspases, may also regulate autophagy-mediated cell death. Atg5 forms a ubiquitin-like conjugation system with Atg12 to enhance the elongation of the autophagosome membrane during vacuole formation [33]. Following several proapoptotic signals, Atg5 can be cleaved and translocated from the cytosol to the mitochondria in a truncated form to activate caspase-dependent apoptosis by interacting with Bcl-X_L_ and promoting cytochrome c release [34]. The autophagic function of Beclin-1 is inhibited by the interaction of Bcl-2 via the Beclin-1–Bcl-2 complex. Bcl-2 acts as a potent antiapoptotic molecule regulating Beclin-1-mediated autophagy and cell death. Moreover, Bcl-2 prevents autophagy-mediated cell death by inhibiting calcium release from the endoplasmic reticulum through Ca^2+^/calmodulin-dependent kinase β and AMP-activated protein kinase activation by calcium, leading to inhibition of mTOR signaling that triggers autophagy [35]. Bcl-2 also negatively regulates autophagy by binding with Beclin-1 through a BH3 domain to interfere with the beclin-1-mediated Type III PI3K complex that plays a role in the formation of autophagic vesicles [36]. However, phosphorylation of Bcl-2 disrupts its interaction with Beclin-1, promoting autophagy by releasing Beclin-1. Therefore, phosphorylation of Bcl-2 can induce apoptosis [33]. Furthermore, signals that promote autophagy or BH3 domain-competitive displacement of Beclin-1 from Bcl-2 by other BH3-containing proteins may lead to the release of Beclin-1 from the Beclin-1–Bcl-2 complex [37]. For example, under starvation stress, c-Jun *N*-terminal protein kinase 1 (JNK1) induces phospho-Bcl-2 and triggers Bcl-2 dissociation from Beclin-1, thereby inducing autophagy-mediated cell death [38].

In the present study, total Bcl-2 levels and Beclin-1–Bcl-2 complex formation decreased upon treatment of colon cancer cells with the KRG extract, both indicating the participation of the Bcl-2 signaling pathway in the KRG extract-induced autophagy-mediated cell death. Moreover, because the KRG extract increased phospho-Bcl-2 levels in a time-dependent manner, it may be assumed that phosphorylated Bcl-2 is linked to apoptosis-mediated cell death, resulting in simultaneous activation of both these processes upon treatment with the KRG extract.

In addition to their molecular connections, apoptosis and autophagy-mediated cell deaths are also regulated by the mitochondria, which function as a switch between them. Briefly, autophagy is induced under conditions of mild mitochondrial stress, whereas apoptosis occurs upon moderate mitochondrial stress caused by the dissociation of proapoptotic factors from the mitochondria [9]. There is a complicated interaction between ROS and autophagy-mediated cell death pathways, which are critical regulators of autophagy-mediated cell death in response to cellular stress. Mitochondrial ROS modulates autophagy during starvation [33] and causes autophagy by inducing nerve growth factor deprivation in sympathetic neurons [34]. It has been reported that ROS modulates the process of autophagy and apoptosis through increasing phospho-Bcl-2 (Ser70) and phospho-Beclin-1 (Thr119) [39]. Aflatoxin B2-generated mitochondrial ROS in hepatocytes causes PI3K/Akt/mTOR-mediated autophagy [40]. In our system, staining of cells with DHR 123, which is a dye used for detecting mitochondrial ROS, revealed that the KRG extract increased ROS generation in the mitochondria of colon cancer cells.

Pre-treatment with NAC (an inhibitor of ROS) significantly rescued the mitochondria from ROS-induced damage (i.e., fewer acridine orange-positive cells), which suggested that autophagy-mediated cell death in colon cancer cells was induced by the KRG extract via increased mitochondrial ROS-mediated cell death. Furthermore, ROS, as a cell death stimulus, also activates the intrinsic or mitochondrial apoptotic pathway by disrupting mitochondrial membrane potential and releasing intermembrane proteins, which are involved in the downregulation of Bcl-2, into the cytosol. Following the formation of the apoptosome, cleaved caspase-9 activates caspase-3, an apoptosis executor, leading to apoptosis [41]. As a result, the increased cleaved caspase-9 and caspase-3 levels showed that the KRG extract exhibited cytotoxicity by activating the intrinsic apoptosis pathway simultaneously with autophagic cell death.

**Figure 6:**
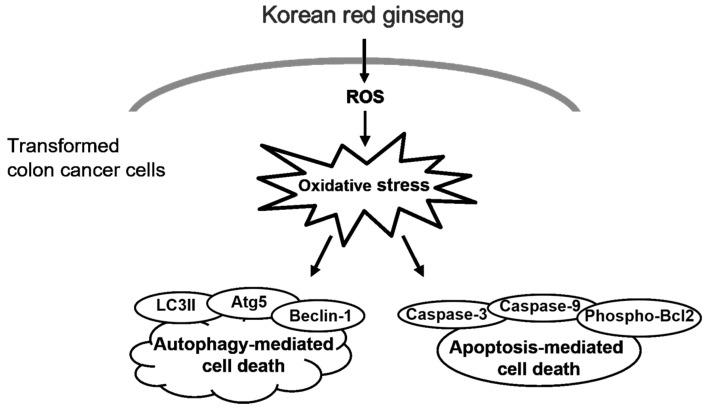
KRG extract induces cell death through oxidative stress. KRG extract-induced ROS generation promotes autophagy- and apoptosis-mediated cell death via activation of phospho-Bcl-2, caspase-3, and caspase-9, as well as Atg5, Beclin-1, and LC3 II.

In conclusion, our novel findings indicate that the KRG extract caused cytotoxicity through autophagy- and apoptosis-mediated cell death in human colon cancer cells by inducing mitochondrial ROS generation and by increasing the expression of Atg5, Beclin-1, phospho-Bcl-2, active caspase-9, and caspase-3 (Figure 6).

## 5. Conclusions

KRG induces autophagy- and apoptosis-mediated cell death via mitochondrial ROS generation, and its administration may inhibit colon carcinogenesis.

## Data Availability

The data presented in this study are available on request from the corresponding author.

## References

[1] Kubczak M., Szustka A., Rogalińska M. (2021). Molecular targets of natural compounds with anti-cancer properties. Int. J. Mol. Sci..

[2] Bata N., Cosford N.D.P. (2021). Cell survival and cell death at the intersection of autophagy and apoptosis: Implications for current and future cancer therapeutics. ACS Pharmacol. Transl. Sci..

[3] Abbas R., Larisch S. (2020). Targeting XIAP for promoting cancer cell death-the story of ARTS and SMAC. Cells.

[4] Gonçalves A.C., Richiardone E., Jorge J., Polónia B., Xavier C.P., Salaroglio I.C., Riganti C., Vasconcelos M.H., Corbet C., Sarmento-Ribeiro A.B. (2021). Impact of cancer metabolism on therapy resistance—Clinical implications. Drug Resist. Updat..

[5] Silva V.R., Neves S.P., Santos L.S., Dias R.B., Bezerra D.P. (2020). Challenges and therapeutic opportunities of autophagy in cancer therapy. Cancers.

[6] Jiang W., Chen X., Ji C., Zhang W., Song J., Li J., Wang J. (2021). Key regulators of autophagosome closure. Cells.

[7] Chen C., Gao H., Su X. (2021). Autophagy-related signaling pathways are involved in cancer. Exp. Ther. Med..

[8] Rahman M.A., Rhim H. (2017). Therapeutic implication of autophagy in neurodegenerative diseases. BMB Rep..

[9] Jung S., Jeong H., Yu S.W. (2020). Autophagy as a decisive process for cell death. Exp. Mol. Med..

[10] Li X., He S., Ma B. (2020). Autophagy and autophagy-related proteins in cancer. Mol. Cancer.

[11] Luo Z., Xu X., Sho T., Zhang J., Xu W., Yao J., Xu J. (2019). ROS-induced autophagy regulates porcine trophectoderm cell apoptosis, proliferation, and differentiation. Am. J. Physiol. Cell Physiol..

[12] Xu L., Fan Q., Wang X., Zhao X., Wang L. (2016). Inhibition of autophagy increased AGE/ROS-mediated apoptosis in mesangial cells. Cell Death Dis..

[13] Qiu Y., Li C., Wang Q., Zeng X., Ji P. (2018). Tanshinone IIA induces cell death via Beclin-1 dependent autophagy in oral squamous cell carcinoma SCC-9 cell line. Cancer Med..

[14] Zhang X.J., Jia S.S. (2016). Fisetin inhibits laryngeal carcinoma through regulation of AKT/NF-κB/mTOR and ERK1/2 signaling pathways. Biomed. Pharmacother..

[15] Baek K.S., Yi Y.S., Son Y.J., Jeong D., Sung N.Y., Aravinthan A., Kim J.H., Cho J.Y. (2017). Comparison of anticancer activities of korean red ginseng-derived fractions. J. Ginseng Res..

[16] Bae J.K., Kim Y.J., Chae H.S., Kim D.Y., Choi H.S., Chin Y.W., Choi Y.H. (2017). Korean red ginseng extract enhances paclitaxel distribution to mammary tumors and its oral bioavailability by P-glycoprotein inhibition. Xenobiotica.

[17] Hyun S.H., Kim S.W., Seo H.W., Youn S.H., Kyung J.S., Lee Y.Y., In G., Park C.K., Han C.K. (2020). Physiological and pharmacological features of the non-saponin components in korean red ginseng. J. Ginseng Res..

[18] Sharma J., Goyal P.K. (2015). Chemoprevention of chemical-induced skin cancer by *Panax ginseng* root extract. J. Ginseng Res..

[19] Kim E.J., Kwon K.A., Lee Y.E., Kim J.H., Kim S.H., Kim J.H. (2018). Korean red ginseng extract reduces hypoxia-induced epithelial-mesenchymal transition by repressing NF-κB and ERK1/2 pathways in colon cancer. J. Ginseng Res..

[20] Lee S., Park J.M., Jeong M., Han Y.M., Go E.J., Ko W.J., Cho J.Y., Kwon C.I., Hahm K.B. (2016). Korean red ginseng ameliorated experimental pancreatitis through the inhibition of hydrogen sulfide in mice. Pancreatology.

[21] Ittiudomrak T., Puthong S., Roytrakul S., Chanchao C. (2019). Alpha-mangostin and apigenin induced cell cycle arrest and programmed cell death in SKOV-3 ovarian cancer cells. Toxicol. Res..

[22] von Muhlinen N. (2019). Methods to measure autophagy in cancer metabolism. Methods Mol. Biol..

[23] Ağuş H.H., Yilmaz S., Şengöz C.O. (2019). Crosstalk between autophagy and apoptosis induced by camphor in *Schizosaccharomyces pombe*. Turk. J. Biol..

[24] Zhao Y.G., Liu N., Miao G., Chen Y., Zhao H., Zhang H. (2018). The ER contact proteins VAPA/B interact with multiple autophagy proteins to modulate autophagosome biogenesis. Curr. Biol..

[25] Park Y.S., Kwon Y.J., Chun Y.J. (2017). CYP1B1 activates Wnt/β-catenin signaling through suppression of Herc5-mediated ISGylation for protein degradation on β-catenin in HeLa cells. Toxicol. Res..

[26] Lee S.M., Bae B.S., Park H.W., Ahn N.G., Cho B.G., Cho Y.L., Kwak Y.S. (2015). Characterization of Korean Red Ginseng (Panax ginseng Meyer): History, preparation method, and chemical composition. J. Ginseng Res..

[27] Yun T.K. (2001). Panax ginsengda non-organ-specific cancer preventive?. Lancet Oncol..

[28] Shin B.K., Kwon S.W., Park J.H. (2015). Chemical diversity of ginseng saponins from Panax ginseng. J. Ginseng Res..

[29] Hong H., Baatar D., Hwang S.G. (2021). Anticancer activities of ginsenosides, the main active components of ginseng. Evid. Based Complement. Alternat. Med..

[30] Yuan H.D., Kim D.Y., Quan H.Y., Kim S.J., Jung M.S., Chung S.H. (2012). Ginsenoside Rg2 induces orphan nuclear receptor SHP gene expression and inactivates GSK3beta via AMP-activated protein kinase to inhibit hepatic glucose production in HepG2 cells. Chem. Biol. Interact..

[31] Li N., Liu B., Dluzen D.E., Jin Y. (2007). Protective effects of ginsenoside Rg2 against glutamate-induced neurotoxicity in PC12 cells. J. Ethnopharmacol..

[32] Jeon H., Jin Y., Myung C.S., Heo K.S. (2021). Ginsenoside-Rg2 exerts anti-cancer effects through ROS-mediated AMPK activation associated mitochondrial damage and oxidation in MCF-7 cells. Arch. Pharm. Res..

[33] Yao C.W., Kang K.A., Piao M.J., Ryu Y.S., Fernando P.M.D.J., Oh M.C., Park J.E., Shilnikova K., Na S.Y., Jeong S.U. (2017). Reduced autophagy in 5-fluorouracil resistant colon cancer cells. Biomol. Ther..

[34] Tsapras P., Nezis I.P. (2017). Caspase involvement in autophagy. Cell Death Differ..

[35] Liu X., Hu X., Kuang Y., Yan P., Li L., Li C., Tao Q., Cai X. (2017). BCLB, methylated in hepatocellular carcinoma, is a starvation stress sensor that induces apoptosis and autophagy through the AMPK-mTOR signaling cascade. Cancer Lett..

[36] Chiang W.C., Wei Y., Kuo Y.C., Wei S., Zhou A., Zou Z., Yehl J., Ranaghan M.J., Skepner A., Bittker J.A. (2018). High-throughput screens to identify autophagy inducers that function by disrupting beclin 1/Bcl-2 binding. ACS Chem. Biol..

[37] Menon M.B., Dhamija S. (2018). Beclin 1 phosphorylation—At the center of autophagy regulation. Front. Cell Dev. Biol..

[38] Ke D., Ji L., Wang Y., Fu X., Chen J., Wang F., Zhao D., Xue Y., Lan X., Hou J. (2019). JNK1 regulates RANKL-induced osteoclastogenesis via activation of a novel Bcl-2-Beclin1-autophagy pathway. FASEB J..

[39] Chen Y., Zhang W., Guo X., Ren J., Gao A. (2019). The crosstalk between autophagy and apoptosis was mediated by phosphorylation of Bcl-2 and beclin1 in benzene-induced hematotoxicity. Cell Death Dis..

[40] Chen B., Li D., Li M., Li S., Peng K., Shi X., Zhou L., Zhang P., Xu Z., Yin H. (2016). Induction of mitochondria-mediated apoptosis and PI3K/Akt/mTOR-mediated autophagy by aflatoxin B2 in hepatocytes of broilers. Oncotarget.

[41] Wu C.C., Bratton S.B. (2017). Caspase-9 swings both ways in the apoptosome. Mol. Cell. Oncol..

