# Correction: Kang et al. Anticolon Cancer Effect of Korean Red Ginseng via Autophagy- and Apoptosis-Mediated Cell Death. *Nutrients* 2022, *14*, 3558

**DOI:** 10.3390/nu15234872

**Published:** 2023-11-22

**Authors:** Kyoung Ah Kang, Cheng Wen Yao, Mei Jing Piao, Ao Xuan Zhen, Pincha Devage Sameera Madushan Fernando, Herath Mudiyanselage Udari Lakmini Herath, Seung Eun Song, Suk Ju Cho, Jin Won Hyun

**Affiliations:** 1 Department of Biochemistry, College of Medicine, Jeju National University, Jeju 63243, Republic of Korea; 2 Jeju Research Center for Natural Medicine, Jeju National University, Jeju 63243, Republic of Korea; 3 Department of Anesthesiology, Jeju National University Hospital, College of Medicine, Jeju National University, Jeju 63241, Republic of Korea

## Error in Figure

In the original publication [1], there was a mistake in the published version of Figure 2e, the authors uploaded the wrong image during final proofreading. In the original version, Figure 2e displays a Western blot image about apoptosis. However, it should display a Western blot about autophagy-related proteins. In the correction, it has been replaced. The corrected version of Figure 2e appears below. 


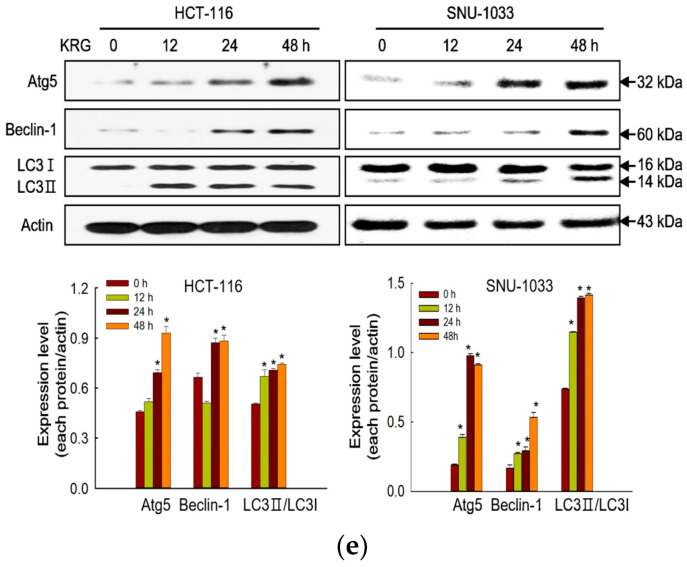


The authors apologize for any inconvenience caused and state that the scientific conclusions are unaffected. This correction was approved by the Academic Editor. The original publication has also been updated.
